# A Proteochemometric Model for Ligands of the SLC5 Transporter Family

**DOI:** 10.1002/ardp.70183

**Published:** 2026-01-16

**Authors:** Martin Juhás, Gerhard Ecker

**Affiliations:** ^1^ Faculty of Pharmacy in Hradec Králové Charles University Hradec Králové Czech Republic; ^2^ Faculty of Science University of Hradec Králové Hradec Králové Czech Republic; ^3^ Department of Pharmaceutical Sciences University of Vienna Vienna Austria

**Keywords:** activity prediction, proteochemometric modeling, SLC5, solute carrier family

## Abstract

The SLC5 family of solute carriers is of significant interest for drug development due to its role in many disease processes. Building on the recent elucidation of SGLT2's structure, we developed a proteochemometric model for SLC5 inhibitors in order to gain information on selectivity‐driving amino acids in the binding site. Ensemble‐based algorithms, namely random forest (RF) and gradient‐boosted trees, proved the best suited for the task reaching high accuracy in both activity and selectivity predictions with Morgan circular fingerprints and Z‐scales for ligand and protein features, respectively. Inclusion of protein sequence as input parameters for the PCM modeling allowed identification of Leu286 in hSLGT2 as a new potential key binding site residue crucial for selectivity. Furthermore, the PCM model also performed well in predicting the effect of single‐point mutations at hSGLT2 on the binding affinity of empagliflozin. The obtained models are available in the form of a Jupyter notebook.

## Graphical abstract

A novel proteochemometric model was developed to predict the activity and selectivity of SLC5 family inhibitors. The model's performance was validated using 10‐fold cross‐validation on publicly available datasets achieving *Q* ^2^ = 0.79 and MSE < 0.32. Detailed analysis of the results enabled the identification of a new, previously unrecognized selectivity‐determining residue in the SLC5 family, offering novel insights for SLC5‐targeted drug design.

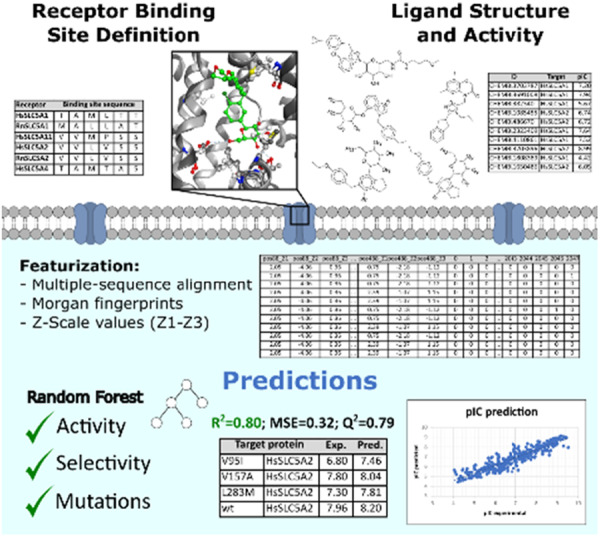

## 1 Introduction

Solute carrier transporters (SLCs) are one of the largest superfamilies of proteins facilitating substrate movement across cellular membranes, predominantly involved in cellular uptake [1, 2]. To this date, the human SLC superfamily is organized into 66 families based on sequence similarity and transport mechanisms [3], comprising passive and secondary active transporters. Function‐wise, the SLC families are diverse and transport a wide range of organic and inorganic molecules, including hormones, neurotransmitter, amino acids, glucose, and xenobiotics (for reviews, see references [1, 2, 4, 5, 6, 7]).

SLC transporters are involved in multiple pharmacological and toxicological processes, and defects in some members have been implicated in severe disorders like amyotrophic lateral sclerosis (ALS) or diabetes mellitus (DM) [2, 6]. Although roughly 30% of the SLC‐transporter are still orphan [8], several members comprise versatile targets in drug development. These include, among others, the GABA transporter GAT1 for epilepsy, the serotonin transporter SERT for depression, and the sodium‐glucose co‐transporter SGLT2 (SLC5A2) for DM. SGLT2 is expressed in the kidney and inhibited by a drug class called gliflozins, leading to improved glucose metabolism in DM patients. However, the favorable effects of the SGLT2‐inhibition seem to go beyond the treatment of DM as observed, for example, in patients with heart failure [9, 10]. Recently, dual inhibition of SGLT1 and SGLT2 has been reinvestigated for the treatment of other glucose‐linked conditions, thus potentially uncovering other positive effects of SLGT2 inhibition in the future [11, 12]. Further drug development efforts will be noticeably improved due to a very recent high‐quality co‐crystal structure of SGLT2 with empagliflozin (PDB ID: 7VSI [13]), which was also used in this study.

Quantitative structure–activity relationship (QSAR) is a well‐established method in computational drug design and the method of choice when structural information for the target is missing. However, traditional QSAR works best on a relatively small chemical space, commonly on a congeneric series of compounds, and is limited to one target per model. These limitations are overcome by proteochemometric modeling (PCM), which simultaneously considers ligands as well as their targets in one model. Thus, PCM uses a more complex and more detailed feature space and can produce more reliable models compared with traditional QSAR [14]. Another advantage of PCM is that by introducing features of the targets, it is not limited to one target only. On the condition that targets’ features of interest are different (sequences or e.g., residues in the binding sites), PCM modeling can be used to study selectivity of ligands across a set of targets, and potentially extrapolate toward new ligands and ideally also toward homologous targets [14]. Furthermore, PCM also allows the use of other organisms' data for direct extrapolation, for example, activities on rat can be used for extrapolation to humans [15]. Thus, PCM has already been applied on various targets, including, for example, also lead optimizations as a part of late‐stage preclinical development [14, 16, 17, 18, 19].

PCM modeling on SLC5 family members had already been successfully attempted by Burggraaff et al. [20], who aimed to discover new inhibitors of hSLGT1. In their work, a mixture of public domain (ChEMBL v.23) and in‐house data were used to produce a classification model, which was validated experimentally, identifying 30 notable inhibitors of hSGLT1 out of 77 classified hits.

However, at that time, the SLC5 members were lacking any experimental (co)crystal structures, so the PCM modeling lacked any structure‐based information, like the exact binding site defined based on the recent hSGLT2 cocrystal [13]. Thus, in this study, we developed a regression‐based PCM model using data from ChEMBL v.30, focusing on the interpretation of the model with respect to residues influencing transporter selectivity. Descriptor importance ranking of the final model allowed identification of Leu286 in hSLGT2 as a new potential key binding site residue important for ligand‐transporter selectivity. Furthermore, the PCM model also allowed to predict the effect of single‐point mutations at hSGLT2 on the binding affinity of empagliflozin.

## 2 Results and Discussion

**Figure 1:**
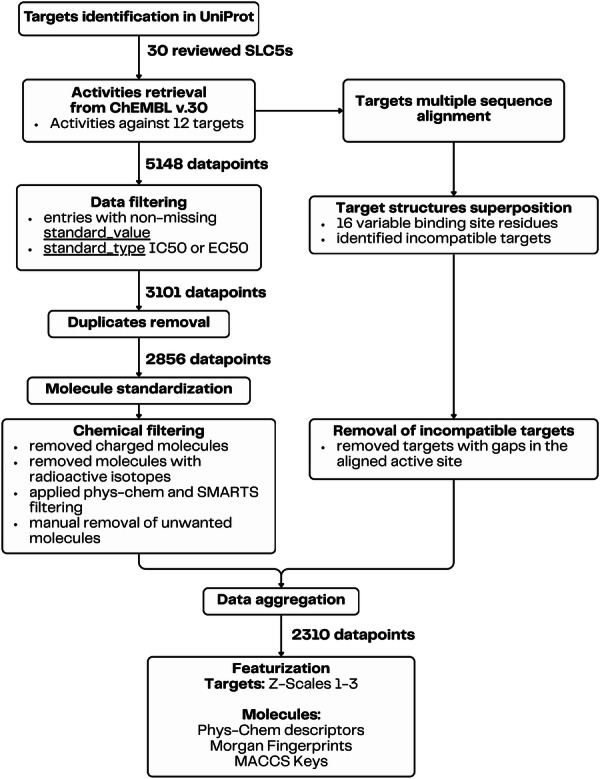
Overall worflow of the data preparation of molecules and targets.

An overall workflow of the data preparation is visualized in Figure 1. Full details of the appropriate steps are described in Section 4.

### 2.1 Data Retrieval

In total, 30 reviewed SLC5 genes were identified in Uniprot for human, mouse, and rat. However, only 12 were also found in ChEMBL v.30 [21], and these are presented in Table 1. The sequences of all 12 targets were aligned using the COBALT web service (https://www.ncbi.nlm.nih.gov/tools/cobalt/re_cobalt.cgi) and processed as described in Section 4.

**Table 1:** Uniprot accession codes and ChEMBL target IDs of chosen SLC5 transporters.

Uniprot	Chembl target ID	Organism	SLC5 subtype
P13866	CHEMBL4979	Homo sapiens (Human)	SLC5A1
Q9NY91	CHEMBL1770047	Homo sapiens (Human)	SLC5A4
Q9GZV3	CHEMBL4507	Homo sapiens (Human)	SLC5A7
P31639	CHEMBL3884	Homo sapiens (Human)	SLC5A2
Q8WWX8	CHEMBL1744524	Homo sapiens (Human)	SLC5A11
Q923I7	CHEMBL1075302	Mus musculus (Mouse)	SLC5A2
Q8BGY9	CHEMBL3013	Mus musculus (Mouse)	SLC5A7
O70247	CHEMBL2176793	Rattus norvegicus (Rat)	SLC5A6
Q63008	CHEMBL2331047	Rattus norvegicus (Rat)	SLC5A5
P53792	CHEMBL4316	Rattus norvegicus (Rat)	SLC5A2
Q9JMD7	CHEMBL5797	Rattus norvegicus (Rat)	SLC5A7
P53790	CHEMBL5374	Rattus norvegicus (Rat)	SLC5A1

**Figure 2:**
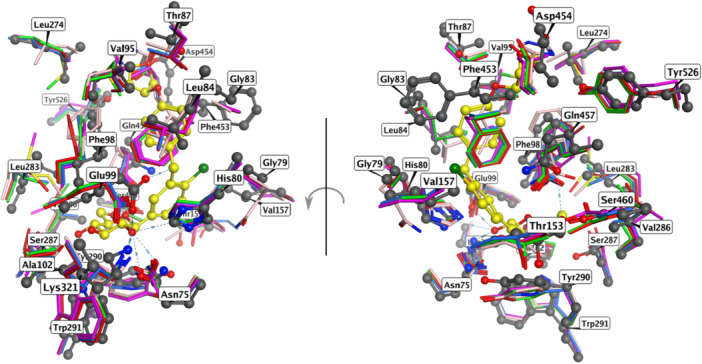
Final alignment of the residues used. Residue numbers correspond to the PDB ID: 7VSI, yellow ball‐and‐stick model is the cocrystallized ligand empagliflozin. Color coding of the proteins: gray: PDB ID: 7VSI; red: AF‐P13866‐F1‐model_v2; green: AF‐P53790‐F1‐model_v2; blue: AF‐P53792‐F1‐model_v2; violet: AF‐Q8WWX8‐F1‐model_v2; beige: AAF‐Q9NY91‐F1‐model_v2. The Figure was generated by MOE 2022.02.

Based on the obtained alignment and subsequent superposition of the SGLT‐2 cocrystal (PDB ID: 7VSI) and AlphFold2 structures, we identified 16 positions in the binding sites that were variable (different amino acids in at least one studied protein), and thus were used for the PCM modeling. The superposition of the selected structures is presented in Figure 2, residues are listed in Table 2.

**Table 2:** Final binding site residues chosen for the modeling. Numbering of the amino acids is based on the appropriate UniProt sequence. For reference, COBALT alignment position and PDB ID: 7VSI position is also included.

		PDB ID: 7VSI	79	87	95	98	102	153	157	274	283	286	287	290	453	454	457	460
		COBALT MSA	88	96	106	109	113	164	168	300	309	312	313	316	481	482	485	488
Target	UniProt ID	ChEMBL ID																
HsSLC5A1	P13866	CHEMBL4979	G82	T90	I98	F101	A105	T156	A160	L274	M283	L286	T287	Y290	F453	D454	Q457	T460
RnSLC5A1	P53790	CHEMBL5374	G82	T90	M98	F101	A105	T156	A160	M274	L283	L286	A287	Y290	F453	D454	Q457	T460
HsSLC5A11	Q8WWX8	CHEMBL1744524	G82	S90	V98	Y101	G105	T156	V160	L269	M278	P281	S282	Y285	F448	I449	Q452	S455
HsSLC5A2	P31639	CHEMBL3884	G79	T87	V95	F98	A102	T153	V157	L274	L283	V286	S287	Y290	F453	D454	Q457	S460
RnSLC5A2	P53792	CHEMBL4316	G77	T85	V93	F96	A100	T151	V155	L272	L281	V284	S285	H288	F451	D452	Q455	S458
HsSLC5A4	Q9NY91	CHEMBL1770047	N82	T90	T98	F101	S105	L156	A160	I274	M283	T286	A287	Y290	I453	H454	E457	S460

### 2.2 ChEMBL Data Processing

In total, ChEMBL v30 contained 5148 activities datapoints (including possible duplicates) for the 12 SLC5s. The dataset was manually curated and filtered to obtain bioactivity value (pIC50) for each compound‐target pair. For a detailed description of the data processing, please see Section 4.

Based on the multiple sequence alignment, datapoints from RnSLC5A5 and HsSLC5A7 were removed as they contained gaps at positions of the binding site. Upon activity data processing, three additional targets got eliminated as they contained permanently charged molecules (MmSLC5A7), missing standard_values (RnSLC5A6), or did not contain IC50 or EC50 values (RnSLC5A7). Finally, datapoints from MmSLC5A2 were removed as MmSLC5A2 and HsSLC5A2 had the same residues in the active site, but different activities for the same compounds were noted, which would confuse PCM modeling. The final dataset contained 2310 datapoints representing 1734 unique molecules and activities against six targets. Out of these, 548 molecules had pIC50 values for more than one target and thus represented the most valuable part of the dataset from the PCM perspective. A detailed datapoint distribution based on targets is presented in Table 3.

**Table 3:** Final distribution of datapoints in the dataset for each target.

UniProt ID	ChEMBL ID	Target	Number of datapoints
**P31639**	CHEMBL3884	HsSLC5A2	1276
**P13866**	CHEMBL4979	HsSLC5A1	983
**Q9NY91**	CHEMBL1770047	HsSLC5A4	26
**P53792**	CHEMBL4316	RnSLC5A2	13
**P53790**	CHEMBL5374	RnSLC5A1	9
**Q8WWX8**	CHEMBL1744524	HsSLC5A11	3

**Figure 3:**
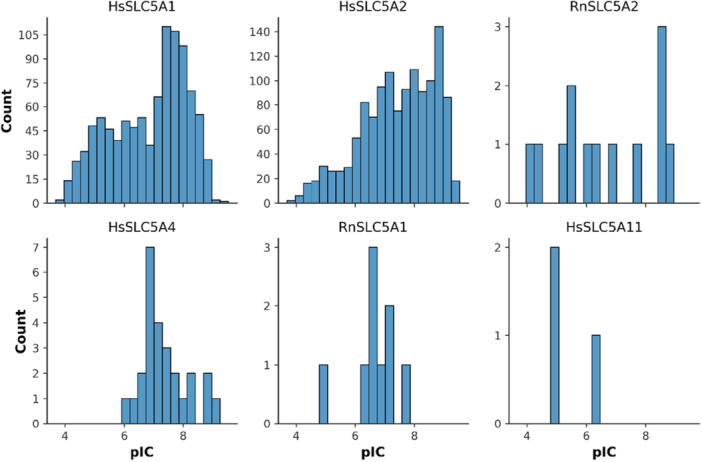
Distribution of the pIC50 values in the final dataset per target.

As can be seen in Table 3 and also from the distribution of the pIC50 values in Figure 3, the obtained dataset was not uniform from the target perspective and biased toward more active compounds. This is an unfortunate problem related to working with public datasets, such as the ChEMBL database [22].

### 2.3 Machine Learning Modeling

A wide range of ligand features have been investigated in PCM modeling with various degree of success [14]. In this study, we used the three most common: Physico‐chemical descriptors, Morgan fingerprints, and MACCSkeys. For details, please refer to Section 4. For encoding the different proteins in the data matrix, we focused on the Z3‐scales, as they not only allow us to trace back which amino acid(s) in the binding site drive ligand selectivity, but also provide information on the respective physicochemical property. Of course, if the main intention is to build a reasonable machine learning model, also, for example, one‐hot encoding usually works quite well.

The training set and test set were obtained by a target‐based stratified split (70/30). Final distribution based on the target is presented in Table 4.

**Table 4:** Final distribution of datapoints in the training set and test set by target.

Target	UniProt ID	ChEMBL ID	Training set (*N* = 1617)	Test set (*N* = 693)
**HsSLC5A1**	P13866	CHEMBL4979	688	295
**RnSLC5A1**	P53790	CHEMBL5374	7	2
**HsSLC5A11**	Q8WWX8	CHEMBL1744524	2	1
**HsSLC5A2**	P31639	CHEMBL3884	893	383
**RnSLC5A2**	P53792	CHEMBL4316	9	4
**HsSLC5A4**	Q9NY91	CHEMBL1770047	18	8

Many machine learning algorithms showed promising results in the PCM; among the most successful were partial least squares (PLS), support vector machine (SVM), random forest (RF), and neural networks (NN) [14]. Here, we investigated only the “classical” machine learning algorithms: SVM, RF, and gradient boosted trees (hereby referred to as XGB based on the used implementation XGBoost [23]).

**Figure 4:**
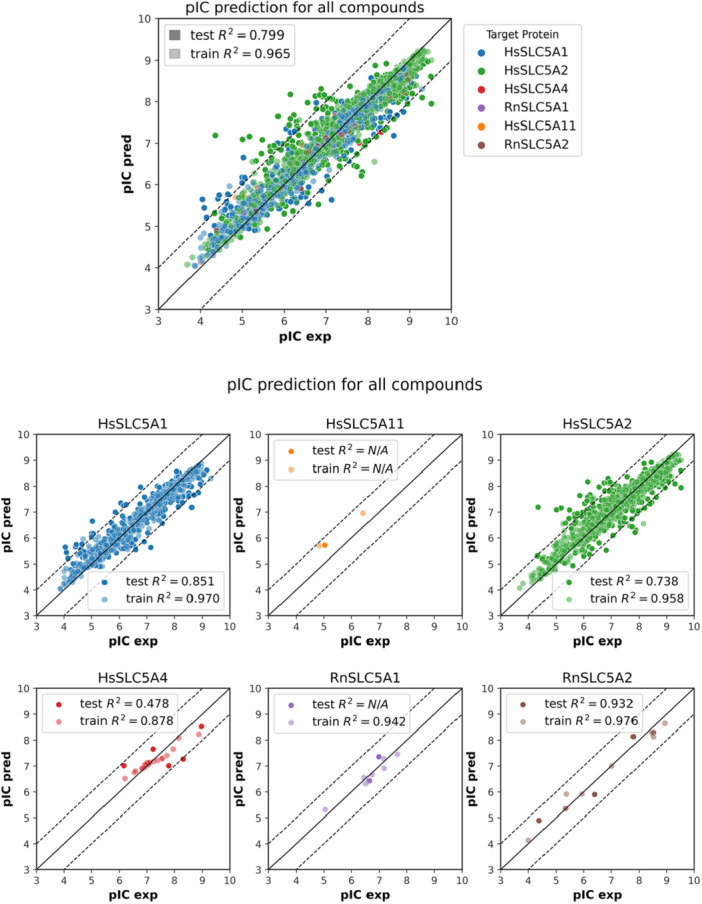
Model performance in pIC prediction in all compounds. Data per target are presented. Dotted lines represent an error of +/− 1 pIC50 unit.

SVM has previously been successfully applied in PCM, and it also worked well in this study [14, 24]. Best predictions were obtained with physico‐chemical descriptors (*R* ^2^ = 0.76 for the final model using 49 phys‐chem features, see Section 4), closely followed by MACCSkeys (*R* ^2^ = 0.75). Despite overall good performance, there were more strong outliers (pICexp−pICpred>2, see below) in the SVM models compared with the later investigated ensemble methods. Morgan fingerprints in SVM achieved the lowest performance with *R* ^2^ = 0.71. Results obtained for all models are presented in Table 5 and in Figure 4.

**Table 5:** Results of the best machine learning models based on the used algorithm.

		SVM	RF	XGB
		*R* ^2^, MSE	*R* ^2^, MSE	*R* ^2^, MSE
Physico‐chemical desc.	Training set	0.88, 0.20	0.97, 0.05	0.98, 0.03
	Test set	0.76, 0.38	0.78, 0.35	0.78, 0.35
	CV (Q^2^)	0.72	0.75	0.77
Morgan circular fingerprint	Training set	0.99, 0.02	0.96, 0.06	0.99, 0.01
	Test set	0.71, 0.46	0.80, 0.32	0.83, 0.27
	CV (Q2)	0.68	0.79	0.79
MACCS keys	Training set	0.92, 0.14	0.95, 0.08	0.94, 0.11
	Test set	0.75, 0.40	0.77, 0.37	0.77, 0.36
	CV (Q^2^)	0.73	0.75	0.76

*Note:* CV Q^2^—10‐fold cross‐validation score.

Ensembles methods such as RF or gradient boosted trees belong to the decision tree‐based algorithms and represent one of the most versatile machine learning algorithms applicable in a wide range of applications. Both RF and XGB performed well, significantly outperforming SVM. The best results were obtained using Morgan circular fingerprints, with *R* ^2^ = 0.80 and *R* ^2^ = 0.82 for RF and XGB, respectively. Physico‐chemical and MACCSKey descriptors scored slightly worse but still outperformed SVM.

Despite XGB achieving (marginally) better overall accuracy, RF models were faster and therefore used for all detailed investigations that follow. If not indicated otherwise, the presented results always apply to the Morgan fingerprints‐based models (random_state=1) as the highest performing ones.

Observing the high accuracy of the RF Morgan fingerprints model, the robustness of the model was further investigated by the DummyRegressor implemented in scikit‐learn. We applied “mean” and “median” prediction strategy; in both cases, the accuracy of the model reached *R* ^2^ ≈ 0.0.

As shown in Figure 4, models for SLC transporter contributing a considerable amount of data (HsSLC5A1 and HsSLC5A2) generally show good performance for the test set, while R2 values for those with only a few data points (HsSLC5A4 and RnSLC5A2) vary considerably. However, this might be due to the different pIC50 ranges covered by the respective data sets rather than their limited size.

#### 2.3.1 Outliers in the Random Forest Models

To investigate the capabilities of the RF PCM models, we trained three randomized models (different initial random_state parameter) for each of the used ligand features using the same train/test split. The goal was to assess the robustness of the ML parameters. The predicted outliers with errors greater than two pIC units (pICexp−pICpred>2, hereby referred to as strong outliers) were gathered and compared within runs and among all models with different ligand featurizations.

Overall, the performance was consistent within the randomized runs, and very few strong outliers were observed. As can be seen in Table 6, the dataset is biased toward active compounds, which is often observed when working with public domain databases like ChEMBL [22]. All the outliers were thus overpredicted, as seen in Tables 7, 8, 9. Good overlap of the outliers was observed within different runs.

**Table 6:** Distribution of pIC50 values in SLC5A2.

SLC5A2	pIC
Mean	7.43
Std	1.24
Min	3.69
25%	6.60
50%	7.59
75%	8.49
Max	9.52

**Table 7:** Outliers observed in models trained using physico‐chemical descriptors.

ChEMBL ID	Structure	pIC50_exp_	pIC50_pred_	Target protein	Times observed
CHEMBL2397443	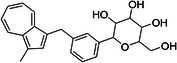	4.36	6.73–6.80–6.92	HsSLC5A2	3
CHEMBL3288757	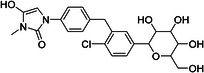	4.05	6.13–6.21–6.22	HsSLC5A2	3

**Table 8:** Outliers in models trained using Morgan fingerprints.

ChEMBL ID	Structure	pIC50_exp_	pIC50_pred_	Target protein	Times observed
CHEMBL1784419	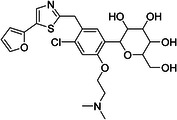	6.15	8.15–8.17–8.18	HsSLC5A2	3
CHEMBL1779206	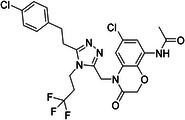	5.01	7.05–7.09–7.16	HsSLC5A2	3
CHEMBL2397443	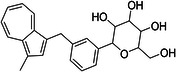	4.36	7.02–7.14–7.19	HsSLC5A2	3
CHEMBL2430318	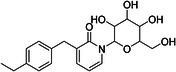	5.18	7.24–7.24	HsSLC5A2	2

**Table 9:** Outliers in models with MACCS keys.

ChEMBL ID	Structure	pIC50_exp_	pIC50_pred_	Target protein	Times observed
CHEMBL1779206	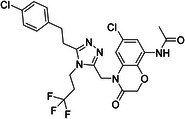	5.01	7.02–7.04–7.10	HsSLC5A2	3
CHEMBL2397443	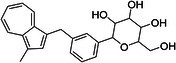	4.36	6.40–6.45–6.47	HsSLC5A2	3
CHEMBL592687	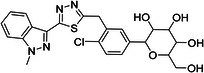	5.48	7.84–7.85–7.85	HsSLC5A2	3
CHEMBL3288757	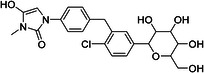	4.04	6.23–6.28–6.31	HsSLCA1	3

The highest number of unique outliers was observed in MACCS‐based models (four, Table 9), but interestingly also in the Morgan fingerprints model (four, Table 8). The lowest number was in the models using Phys‐Chem properties (two, Table 7). This was surprising since fingerprint‐based models overall scored better than Phys‐Chem properties‐based.

One outlier compound appeared in all models, CHEMBL2397443, while two outliers (including CHEMBL2397443) were shared in Morgan and Phys‐Chem (CHEMBL1779206, CHEMBL2397443), and two were shared in MACCS and Phys‐Chem (CHEMBL2397443, CHEMBL3288757). However, upon closer inspection, in most cases the difference of pIC_exp_ and pIC_pred_ was only slightly above the defined cutoff as exemplified by compounds CHEMBL1779206 and CHEMBL1784419 in Morgan models and/or MACCS models, where the pIC50 prediction was approx. 2.1 pIC50 units overpredicted (see Tables 8, 9). Not considering these slight overpredictions, we observed only a handful of outliers.

**Figure 5:**
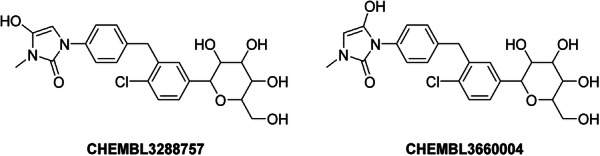
Comparison of chemical structures of compounds CHEMBL3288757 and CHEMBL3660004.

The most notable outlier is CHEMBL3288757, a highly selective SGLT‐1 inhibitor (pIC50 = 6.31). While its SGLT‐1 activity was present in the test set, the SGLT‐2 (inactive) datapoint (pIC50 = 4.05) was in the training set. At first, we thought the overprediction of CHEMBL3288757, which occured in Phys‐Chem and MACCS models, was due to the inability of distinguishing these two targets. However, upon closer inspection, we discovered that this compound comes from a larger congeneric series of compounds containing several activity cliffs, with some of these compounds being in the training set. As an example, we present CHEMBL3660004, a very potent but non‐selective inhibitor of SGLT‐1 and SGLT‐2 (pIC50 = 7.08 and 6.83, respectively) that is a positional isomer of CHEMBL3288757 (Figure 5). Both activities were in the training set, thus this overprediction was due to the presence of an activity cliff that was insufficiently described by some descriptors. Similar activity cliffs were also observed for CHEMBL2397443, which was also observed as an outlier in all models.

Activity cliffs are generally challenging in drug design and present an exceptional challenge for QSAR modeling. However, as investigated, for example, by Tilborg and colleagues [25], use of circular fingerprints seems appropriate for cases where activity‐cliffs are expected, supporting why Morgan fingerprints outperformed other descriptors in the current study.

In the following tables, the chemical structure represents structures after final standardization for the ML modeling, that is, without chirality assigned.

### 2.4 Importance of the Target Residues

Studying selectivity, we were particularly interested in the importance of the chosen pocket residues, that is, the target features. The main rationale was to find which positions (residues) are crucial for predicting the correct pIC50, and could thus potentially be responsible for the selectivity of the inhibitors toward (investigated) isoforms of SLC5s. This approach has already been successfully applied in PCM modeling by others, for example, on serine proteases [26], GPCRs [27], or HDAC [28] receptors, as well as on GABA transporters by our group [29]. Numbering of residues/positions in the following section refers to the position of the residue in the SLGT2, since this can be easily visualized using the crystal structure PDB ID: 7VSI. For reference, we are also including the position in the COBALT MSA. Full mapping of the binding site residues of the investigated targets, including their position in the COBALT MSA is presented in Table 2.

A big advantage of RF, and other decision tree‐based methods, is their natural feature importance awareness and thus ability to rank the features’ impact on the prediction. As Morgan fingerprints had the most accurate predictions, this model was used for the investigations. Little attention was paid to the ligand features since comparison and interpretation of bits in Morgan fingerprints would be a nontrivial matter.

Two different methods for importance investigations were used. In the first one (1), the ranking of the features was obtained directly from scikit‐learn. The disadvantage of this method was that it could not distinguish if other feature(s) could substitute the impact of the feature (i.e., specific amino acid residue represented by the Z‐scale). We tried to mitigate this by the second method (2), doing an exhaustive permutation of all positions, retraining the model with the best obtained parameters (no grid searching) and monitoring the performance.

The feature importances obtained from the trained model are expressed as the mean decrease in impurity of the trees in the RF model, with the higher values meaning higher importance (see feature_importances in scikit‐learn). For our investigations, considering there were 1443 features in the models, we found working with the ranks more illustrative of the features’ importance. Note that by ranking the importances in the ascending order their original meaning is retained; the higher the importance, the higher its rank.

The first method found position 286 (position 312 in COBALT MSA) to be the most important and was thus ranked the highest. More precisely, it was its Z2‐scale value (steric properties). This position was closely followed by positions 95 (with Z1‐scale=lipophilicity and Z3‐scale=electronic properties) and 460 (Z1, Z2, and Z3‐scale). Table 10 shows the mean, median, and standard deviation of the ranks from the top 10 ranked positions within five randomized runs (different random_state parameter). The mean in this case represents the average ranking of the feature as returned by scikit‐learn. Considering that the majority of 1443 descriptors in the model are represented by the bits of the Morgan fingerprints, the rank 1438 of position 286 is significant, that is, the feature is very important for model performance.

**Table 10:** Top 10 best ranking Z‐scales from RF model with Morgan fingerprint descriptors; higher is better (*N* = 5).

PDB ID: 7VSI	COBALT_MSA	Mean	Median		std	
pos286_Z2	pos312_Z2	1438.6	1440.0		2.3	
pos95_Z3	pos106_Z3	1431.4	1432.0		4.8	
pos460_Z1	pos488_Z1	1430.2	1431.0		6.8	
pos460_Z2	pos488_Z2	1425.8	1431.0		10.4	
pos95_Z2	pos106_Z2	1424.2	1427.0		11.0	
pos95_Z1	pos106_Z1	1422.8	1428.0		15.1	
pos287_Z3	pos313_Z3	1422.2	1426.0		14.6	
pos460_Z3	pos488_Z3	1421.8	1420.0		6.8	
pos286_Z1	pos312_Z1	1408.8	1413.0		20.2	
pos286_Z3	pos312_Z3	1408.0	1416.0		19.6	

In the second method, we focused only on the selected amino acid position; Z‐scale values were not distinguished. The same residues as in the first method were identified as important. Due to ignoring the Z‐scales, a slightly different ranking was observed. The following six positions were essential for the model, in the order of decreasing importance: 460 (488 in COBALT MSA), 286 (312 in COBALT MSA), 95 (106 in COBALT MSA), 287 (313 in COBALT MSA), 157 (168 in COBALT MSA), 283 (309 in COBALT MSA). Removal of any of these residues from the feature space strongly deteriorated the performance, effectively reaching the performance of a model where no positions were included (*R* ^2^ ≈ 0.36, *Q* ^2^ ≈ 0.37). When only one position was added back, the best performance was again observed with residue 460 *R* ^2^ = 0.79 (*Q* ^2^ = 0.79), closely followed by 286, 95, 287, 157, and 283, which achieved the worst score, *R* ^2^ = 0.77 (*Q* ^2^ = 0.75). A model containing only the six residues performed equivalently to the model with all selected binding site residues *R* ^2^ = 0.80 (*Q* ^2^ = 0.79), thus showing a relatively small (statistical) impact of other residues toward activity prediction.

The identified important positions and the respective amino acids present in the investigated SLC5 proteins are indicated in Table 11.

**Table 11:** The positions identified by the PCM modeling and the respective amino acids. Numbering based on 7VSI and COBALT alignment is indicated.

PDB ID:7VSI	95	157	283	286	287	460
COBALT MSA	106	168	309	312	313	488
HsSLC5A1	I	A	M	L	T	T
RnSLC5A1	M	A	L	L	A	T
HsSLC5A11	V	V	M	P	S	S
HsSLC5A2	V	V	L	V	S	S
RnSLC5A2	V	V	L	V	S	S
HsSLC5A4	T	A	M	T	A	S

**Figure 6:**
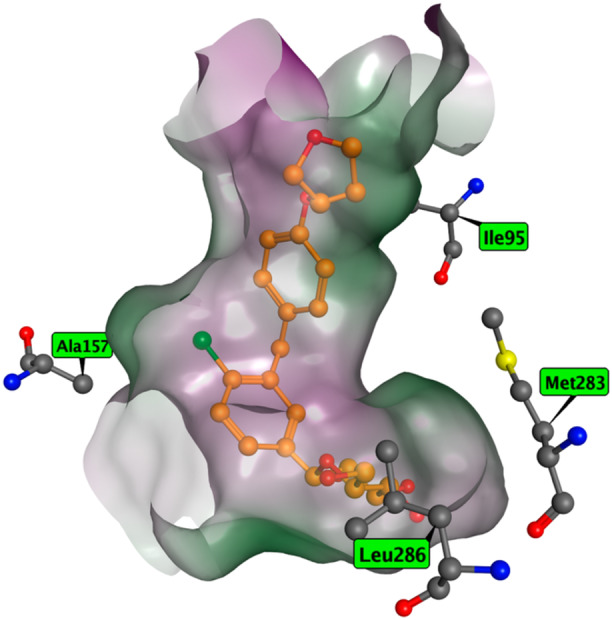
Graphical visualization of position 286 (Leu286 in hSLGT2) and other currently validated point‐mutation analyzed positions leading to lowering of the hSGLT2 activity. (PDB ID: 7VSI).

Yet, a mathematical significance does not necessarily translate to biological relevance. Unfortunately, to our best knowledge, there has been no experimental mutation analysis involving residue 286 that we saw consistently being the most impactful (see Figure 6). However, other impactful positions, notably the close by residues at positions 95, 157, and 283 were experimentally validated by point‐mutation analyses in hSGLT2. The mutation of these residues to the corresponding amino acids from hSGLT1 (V95I, V157A, and L283M) showed reduced inhibitory activity of empagliflozin (highly selective SGLT2 inhibitor) while retaining the glucose absorption of the mutant [13]. We thus conclude that the observed residue 286 could also bear a strong biological significance for the SLC5 family, and perhaps also participate or govern the selectivity of inhibition among its members.

### 2.5 Ability of the Model to Correctly Predict Selectivity

Finally, we have closely analyzed the ability of the model to correctly predict the selectivity of compounds. Out of all 548 unique molecules with activity values on more than one SLC transporter (1124 datapoints in the whole dataset), the test set contained 278 molecules (330 datapoints). The activity prediction of these compounds was satisfactory, overall reaching *R* ^2^ = 0.84, MSE = 0.29, with 92% being within ±1 pIC50% and 70% within ±0.5 pIC50 (test set metrics). Thus, the model was able to successfully predict activity for specific targets on unseen data, despite the compound had multiple, often largely different activities against specific SLC5 isoforms.

**Figure 7:**
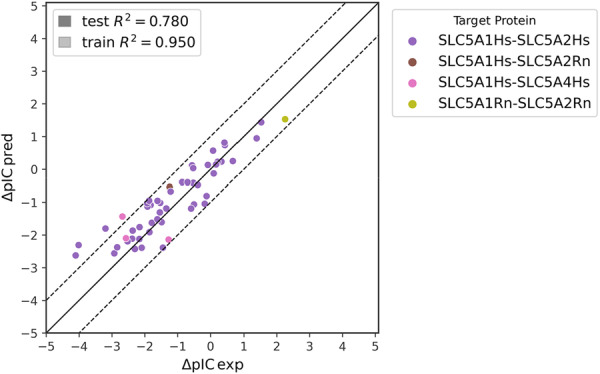
Model preformance for compounds with multiple activities present only in the test set. Dotted lines represent an error of +/‐ 1 pIC50 unit.

To simulate the ability to correctly predict target selectivity, we analyzed a set of 52 compounds that had more than one pIC50 value, but were present only in the test set (i.e., the model could not have learned it from the training set). Selectivity was judged as $\mathrm{pI}C_{\mathrm{diff}}=\;{\left(\mathrm{pI}C_{\mathrm{target}1}-\mathrm{pI}C_{\mathrm{target}2}\right)}_{\text{exp}}-\;{\left(\mathrm{pI}C_{\mathrm{target}1}-\mathrm{pI}C_{\mathrm{target}2}\right)}_{\mathrm{pred}}$, where $\mathrm{pI}C_{\mathrm{diff}}=0$ means the model predicted a compound to be selective against target 1 compared with target 2 with the same magnitude as observed experimentally. The quality of prediction of the pIC50 itself was investigated above. Thus, 92% of the predictions were within ±1% and 58% within ±0.5 *pIC*_*diff*_ (*R* ^2^ = 0.78, MSE = 0.39). As evident from Figure 7, the model tends to overpredict ($\mathrm{pI}C_{\mathrm{pred}}>\mathrm{pI}C_{\text{exp}}$), which is the unfortunate result of the insufficient number of low activity datapoints in ChEMBL.

The presented model was therefore able to predict both the activity and degree of selectivity of the compound‐target pairs with reasonable accuracy and correctness, and none of the observed compounds was identified as a strong outlier (difference in pIC50 > 2).

As a proof of concept, we used the in silico point‐mutated pocket as investigated by Niu et al. [13] for the protein features and predicted the pIC50 of empagliflozin, for which the point‐mutation analyses were available. It is important to note that the mutated proteins' activities were not part of the dataset. As shown in Table 12, the model was able to correctly predict the decreased activity against the mutated SGLT2 compared with the wild type. More rigorous testing would require additional experimental pIC50s resulting from the point mutation analyses, which were not available at the time of this study. Once again, the predictions based on Morgan fingerprint models outperformed the other two descriptors (physico‐chemical and MAACS).

**Table 12:** Prediction of empagliflozin activity against wild‐type and point‐mutated SGLT2 (HsSLC5A2) in the random forest model.

Target protein	Literature value^a^	Phys‐chemical descriptors	Morgan circular fingerprints	MACCS fingerprints
V95I HsSLC5A2	6.80 (157.7)	7.96	7.46	7.74
V157A HsSLC5A2	7.80 (15.84)	8.29	8.04	8.12
L283M HsSLC5A2	7.30 (50.62)	8.06	7.81	8.05
wt HsSLC5A2	7.96 (10.88)	8.38	8.20	8.28

^a^ Values in parentheses represent published EC_50_ in [nM] [13].

### 2.6 Investigation of the Chemical Space and Applicability Domain

It has been previously noted that ChEMBL covers a large but also realistic chemical space [30]. That is the reason why ChEMBL alone or in combination with other databases serve as a starting point for the ligand‐based drug design.

**Figure 8:**
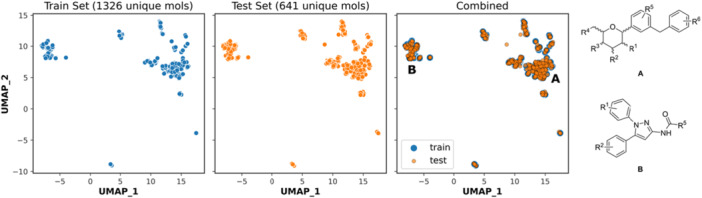
UMAP plot of unique molecules from the training set, test set, and combined.

The chemical space of SLC5 inhibitors, as used in this study, is unfortunately biased by the public unavailability of (in)active data. The most abundant chemotypes present in the dataset, denoted as **A** and **B** in Figure 8, correspond to the aryl‐substituted saccharides (structure **A**) and diaryl‐substituted pyrazoles (structure **B**). In both cases, the substituents R^1‐4^ represent mostly short alkyl, hydroxy, and/or halogen substitution, while R^5,6^ represent mostly a further decorated fragment and/or another (annelated) ring.

**Figure 9:**
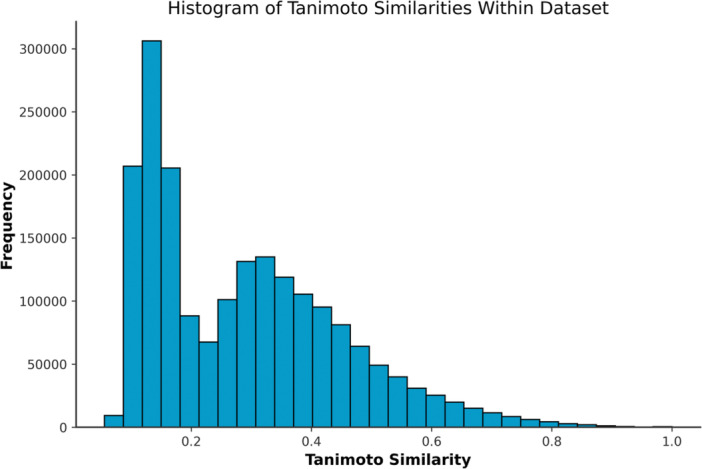
Histogram of whole‐dataset Tanimoto similarities.

Still, as seen in Figure 9, the majority of molecules share only very low whole‐structure similarity with a Tanimoto index < 0.4. We thus believe that there is sufficient chemical variability for the purpose of studying the selectivity of SLC5 inhibitors.

## 3 Conclusions

We have shown that PCM is a valuable method for deeper insights into the selectivity of inhibitors in the SLC5 family. This study produced three machine learning models using traditional algorithms, SVM, RF, and XGB, capable of predicting the activity and, more importantly selectivity of SLC5 inhibitors based on the input structure and receptor pocket residues. The models were validated using cross‐validation methodology and proved accurate, with the best *Q* ^2^ = 0.80. We have shown a potential applicability of the model on predicting selectivity on point‐mutated receptors with qualitatively correct predictions, although additional point‐mutation training data would be required to obtain a quantitative model. By studying the selectivity, we uncovered a new potential binding site residue of interest, possibly responsible for the selectivity of SLC5 inhibitors that could be prioritized in further research of the new SLC5 inhibitors as potential drugs.

## 4 Experimental

All data manipulation was done in Python 3.7 using JupyterLab.

### 4.1 Data Retrieval

Uniprot accession codes for all reviewed human (*Homo sapiens, ID:* 9606), mouse (*Mus musculus, ID: 10090*) and rat (*Rattus norvegicus, ID: 10116*) slc5 genes were gathered manually and translated to ChEMBL target IDs using the REST API (ChEMBL v.30 was used, https://www.ebi.ac.uk/chembl/). Upon identifying available targets in ChEMBL, the activities data were downloaded using the REST API and saved. All REST API calls were done using an inhouse Python script.

### 4.2 Targets Multiple Sequence Alignment

Primary sequences of all targets available in ChEMBL were aligned using the COBALT webservice (https://www.ncbi.nlm.nih.gov/tools/cobalt/re_cobalt.cgi), using default settings. Subsequently, AlphaFold2 [31] structures of all identified proteins without available crystal structure were retrieved and superimposed on the crystal structure of human SGLT2 complexed with empagliflozin (PDB ID: 7VSI). Binding site residues were defined in MOE 2020.09 using the Pocket definition (residues with atoms within 4.5 Å of the co‐crystallized ligand), and the corresponding residues in the other transporter were identified based on the COBALT alignment. Only positions with amino acid variability were considered for PCM modeling. For the proteins with gaps within the chosen positions, AlphaFold2 structures were aligned to PDB ID: 7VSI one‐by‐one based only on the structure in MOE to improve the alignment. If there were still gaps observed, the receptors were excluded. COBALT alignment of the final chosen targets in FASTA format is available in the Supporting Information S1.

### 4.3 Dataset Processing and Molecule Standardization

All datapoints with missing values in Standard Unit, Standard Relation, and Standard Type were deleted. Only datapoints of Standard Type “IC50” or “EC50”, and Standard Unit “=” were retained.

Datapoints coming from the same source (filtered by a combination of molecule ChEMBL ID, target ChEMBL ID, and document ChEMBL ID) and/or having the exact same standard value in various documents (filtered by a combination of molecule ChEMBL ID and target ChEMBL ID) were considered duplicates and were thus merged into one single datapoint. The second filter was applied to treat cases where one document referenced the value of another document.

The data set was manually curated to identify and correct possible errors, for example, incorrect activity values in the ChEMBL database from Xu et al. [32] (document CHEMBL1806485) or incorrect SMILES strings for derivatives from Lee et al. [33] (document CHEMBL1287682). After corrections, the data set was filtered, and only datapoints with IC_50_ or EC_50_ values were considered for further modeling. The biological activities were aggregated into a pIC value pIC=−log(standard_value[nM]/1e9) per available molecule‐target pair.

The SMILES strings (as retrieved from ChEMBL) were standardized and translated to InChIKeys by RDKit (function MolToInchiKey, v2021.03.4). Standardization consisted of cleaning up the molecule by disconnecting metal atoms, reionizing, retaining the parent fragment, neutralizing, removing stereochemistry, generating canonical tautomer, and adding hydrogens. Following code was used:

def standardize_smiles(smi):

m = Chem·MolFromSmiles(smi)
m = rdMolStandardize·Cleanup(m)
m = rdMolStandardize·FragmentParent(m)
m = rdMolStandardize·ChargeParent(m)
Chem·rdmolops·RemoveStereochemistry(m)
te = rdMolStandardize·TautomerEnumerator()
m = te·Canonicalize(m)
m = Chem·AddHs(m)
return m

Molecules having permanent charge or with internally charged atoms (SMARTS: “[#7+]”, “[#7−]”, “[#8+]”, “[#8−]”, “[#16+]”, “[#16−]”, “[#5+]”, “[#5−]”) or contained isotopes (SMARTS: “[2H]”, “[13#6]”, “[17#8]”, “[15#7]”) were removed. Physico‐chemical properties filtering was then applied to remove compounds with MolWt < 150, MolWt > 800 and NumAromaticRings > 5 (functions from RDKit with the same names were used), and SMARTS filtering “protein backbone AND NOT hexose AND NOT pentose” was applied to remove any potential peptides that could possess a different mechanism of action than the small molecules. Following SMARTS were used:

- protein backbone SMARTS pattern: “[$(CN)][$(C( = O)NCC( = O))]”
- hexose SMARTS pattern: “[$(C1(O)COCC(O)C1(O))]”
- pentose SMARTS pattern: “[$(C1(O)COCC1(O))]”

Finally, compounds were checked manually, and compounds containing fluorescent probes (CHEMBL449778, CHEMBL443616, CHEMBL445247) and triphenyl protection (CHEMBL3288753) were removed. The data set was then aggregated based on the InChIKeys. For InChIKeys having less than five activity datapoints for a particular target, the average was taken if values were within SEM < = 0.3, otherwise all activity data for that molecule‐target pair were removed. For InChIKeys with five or more activities, the average was taken.

### 4.4 Investigation of the Chemical Space and Applicability Domain

The chemical space was investigated using da imensionality reduction plot by Uniform Manifold Approximation and Projection for Dimension Reduction (UMAP) [34]. The python package umap‐learn 0.5.3.

First, the embeddings for the training set were obtained by extracting unique molecules from the training set. Note that without this step, there would be instances of the same molecule due to having activity on different targets. Then, the obtained model was applied on the test set to compare the covered chemical space as well as the applicability domain of the PCM models. Compounds were represented by the same Morgan fingerprints as during the featurization, that is, radius=2 and nBits=2048. For the UMAP hyperparameters, “jaccard” metric was used with n_neighbors=50 and min_distance=0.001. Value for the n_neighbors was chosen as best from the tested values of 2, 5, 10, 25, 50, 80, and 100. The obtained model is available with the prediction Jupyter Notebook code in the Supporting Information S2.

### 4.5 Machine Learning Modeling

For the receptors, that is, the binding site residues, the first three Z‐scales described by Sandber and colleagues [35], related to lipophilicity, steric, and electronic properties were used as features.

For ligands, physico‐chemical descriptors, Morgan fingerprints, and MACCS keys were used. The phys‐chem descriptors were calculated from available descriptors in RDKit 2021.03.4. Out of 208 available descriptors, all fragment descriptors (starting with “fr_”) were discarded, and from the rest, descriptors with obvious redundant information were filtered out manually, for example, from MolWt, HeavyAtomMolWt, ExactMolWt, only MolWt was retained. Thus, we obtained 108 features that were used in the initial models; the descriptors are listed in the Supporting Information S2. Later, the features were manually revised. Highly correlated descriptors (absolute of the Pearson coefficient > 0.7) were removed, and from the rest 49 descriptors representing common molecular properties (electronic, lipophilicity, shape, etc.) were handpicked. The list of final descriptors is also provided in the Supporting Information S2. The simpler models were found to perform similarly to those with all physico‐chemical descriptors, and only these models are therefore commented in this article. A vector of 2048 bits with a radius of 2 was used for Morgan circular fingerprints. MACCS keys were calculated as bit vectors. In the case of all descriptors, only non‐constant features were used in model building. No cross terms were used.

The ML modeling was done using algorithms as implemented in scikit‐learn 1.0.2 and xgboost v.1.6.1. The 10‐fold cross‐validation monitored overfitting. Final cross‐validated *R* ^2^, hereby reported as *Q* ^2^, represents the average of the 10‐fold *R* ^2^ values. The performance was judged based on *R* ^2^, *Q* ^2^, and MSE. Optimal hyperparameters for the ML algorithms were obtained by performing a grid search using GridSearchCV as implemented in scikit‐learn. Details for hyperparameter optimization as well as final hyperparameters used for all the models can be found in the Supporting Information S2. SVR, RandomForestRegressor, and XGBRegressor classes were used for the ML modeling. For the SVM models, the data were scaled using StandardScaler function. The r2_score, cross_val_score, and mean_squared_error functions were used to evaluate the model performance, 10‐fold cross‐validation was used. The final plots were done using matplotlib 3.5.2 and seaborn 0.11.2.

## Supporting information

SI_new.

Supplementary_material_rev1.
