# Supplementary material for: A Proteochemometric Model for Ligands of the SLC5 Transporter Family
Source: Arch Pharm (Weinheim). 2026 Jan 16;359(1):e70183. doi: 10.1002/ardp.70183 (PMC12809628; doi:10.1002/ardp.70183)
Supplement: Supplementary file 2 — Supplementary_material_rev1. [file ARDP-359-e70183-s001.pdf]

A proteochemometric model for ligands of the SLC5 transporter family

Martin Juhás[1,2], Gerhard Ecker[3]

[1] Charles University, Faculty of Pharmacy in Hradec Králové, Akademika Heyrovského 1203, 50009 Hradec Králové, Czech Republic
[2] University of Hradec Králové, Faculty of Science, Rokitanského 62, 50003 Hradec Králové, Czech Republic
[3] University of Vienna, Faculty of Science, Josef-Holaubek-Platz 2, 1090 Vienna, Austria

Supplementary Information

Contents

Hyperparameter optimization 2
Final hyperparameters used for the ML models 2
Physico-chemical descriptors 3
All descriptors used in the initial phys-chem descriptor-based models 3
Descriptors used in the final models 3

Hyperparameter optimization

To obtain optimal hyperparameters for the used algorithms, the hyperparameters of each model were optimized. The *SVM models* always used the **rbf** kernel with **gamma** set to scale. Only the regularization parameter **C** and **epsilon** parameter were tuned:

- **C** = [0.1, 1, 10, 20, 50, 80, 100]
- **epsilon** = [0.1, 0.2, 0.4]

In the *random forest models*, the number and depth of created trees as well as number of features to use for tree splitting was tuned, **bootstrap** was used:

- **n_estimators** = [100, 300, 500, 800, 1000]
- **max_depth** = [10, 25, None]
- **max_features** = [0.1, 0.3, 0.5, 0.7, 1.0]

Lastly, in the XGB models, the estimators in XGB were tuned by:

- **n_estimators** = [100, 300, 500],
- **learning_rate** = [0.01, 0.1],
- **max_depth** = [10, None],
- **subsample** = [0.1, 0.4, 0.7, 1.0],
- **colsample_bytree, colsample_bylevel**, **colsample_bynode** = [0.1, 0.4, 0.7, 1.0] (for each)

The rest of the hyperparameters were kept constant based on the default values in scikit-learn v1.0.2 and/or xgboost v1.6.1.

Final hyperparameters used for the ML models

Physico-chemical descriptors:

SVM: 'C': 10, 'epsilon': 0.1, 'gamma': 'scale', 'kernel': 'rbf'

RF: 'bootstrap': True, 'max_depth': None, 'max_features': 0.5, 'max_leaf_nodes': None, 'max_samples': None, 'min_samples_leaf': 1, 'min_samples_split': 2, 'n_estimators': 300

XGB: 'colsample_bylevel': 0.4, 'colsample_bynode': 0.7, 'colsample_bytree': 1.0, 'learning_rate': 0.01, 'max_depth': 10, 'n_estimators': 500, 'subsample': 1.0

Morgan fingerprints:

SVM: 'C': 50, 'epsilon': 0.1, 'gamma': 'scale', 'kernel': 'rbf'

RF: 'bootstrap': True, 'max_depth': 25, 'max_features': 0.1, 'max_leaf_nodes': None, 'max_samples': None, 'min_samples_leaf': 1, 'min_samples_split': 2,'n_estimators': 100

XGB: 'colsample_bylevel': 1.0, 'colsample_bynode': 1.0, 'colsample_bytree': 0.4, 'importance_type': 'gain', 'learning_rate': 0.1, 'max_depth': 10, 'n_estimators': 500, 'subsample': 0.7, 'tree_method': 'exact'

MACCS fingerprints:

SVM: 'C': 10, 'epsilon': 0.1, 'gamma': 'scale', 'kernel': 'rbf'

RF: 'bootstrap': True, 'max_depth': 25, 'max_features': 0.3, 'max_leaf_nodes': None, 'max_samples': None, 'min_samples_leaf': 1, 'min_samples_split': 2, 'n_estimators': 1000

XGB: 'n_estimators': 300, 'learning_rate': 0.1, 'max_depth': None, 'subsample': 0.7, 'colsample_bylevel': 0.7, 'colsample_bynode': 0.7, 'colsample_bytree': 0.4,

Physico-chemical descriptors

All descriptors used in the initial phys-chem descriptor-based models

'MolWt', 'NumValenceElectrons', 'FpDensityMorgan1', 'FpDensityMorgan2', 'FpDensityMorgan3', 'BCUT2D_MWHI', 'BCUT2D_MWLOW', 'BCUT2D_CHGHI', 'BCUT2D_CHGLO', 'BCUT2D_LOGPHI', 'BCUT2D_LOGPLOW', 'BCUT2D_MRHI', 'BCUT2D_MRLOW', 'BalabanJ', 'BertzCT', 'Chi0', 'Chi0n', 'Chi0v', 'Chi1', 'Chi1n', 'Chi1v', 'Chi2n', 'Chi2v', 'Chi3n', 'Chi3v', 'Chi4n', 'Chi4v', 'HallKierAlpha', 'Ipc', 'Kappa1', 'Kappa2', 'Kappa3', 'LabuteASA', 'PEOE_VSA1', 'PEOE_VSA2', 'PEOE_VSA3', 'PEOE_VSA4', 'PEOE_VSA5', 'PEOE_VSA6', 'PEOE_VSA7', 'PEOE_VSA8', 'PEOE_VSA9', 'PEOE_VSA10', 'PEOE_VSA11', 'PEOE_VSA12', 'PEOE_VSA13', 'PEOE_VSA14', 'MolMR', 'SMR_VSA1', 'SMR_VSA2', 'SMR_VSA3', 'SMR_VSA4', 'SMR_VSA5', 'SMR_VSA6', 'SMR_VSA7', 'SMR_VSA8', 'SMR_VSA9', 'SMR_VSA10', 'MolLogP', 'SlogP_VSA1', 'SlogP_VSA2', 'SlogP_VSA3', 'SlogP_VSA4', 'SlogP_VSA5', 'SlogP_VSA6', 'SlogP_VSA7', 'SlogP_VSA8', 'SlogP_VSA9', 'SlogP_VSA10', 'SlogP_VSA11', 'SlogP_VSA12', 'TPSA', 'EState_VSA1', 'EState_VSA2', 'EState_VSA3', 'EState_VSA4', 'EState_VSA5', 'EState_VSA6', 'EState_VSA7', 'EState_VSA8', 'EState_VSA9', 'EState_VSA10', 'EState_VSA11', 'VSA_EState1', 'VSA_EState2', 'VSA_EState3', 'VSA_EState4', 'VSA_EState5', 'VSA_EState6', 'VSA_EState7', 'VSA_EState8', 'VSA_EState9', 'VSA_EState10', 'FractionCSP3', 'RingCount', 'NumAliphaticCarbocycles', 'NumAliphaticHeterocycles', 'NumAliphaticRings', 'NumAromaticCarbocycles', 'NumAromaticHeterocycles', 'NumAromaticRings', 'NumSaturatedCarbocycles', 'NumSaturatedHeterocycles', 'NumSaturatedRings', 'NumHAcceptors', 'NumHDonors', 'NumHeteroatoms', 'NumRotatableBonds'

Descriptors used in the final models

'FpDensityMorgan1', 'FpDensityMorgan2', 'FpDensityMorgan3', 'BCUT2D_MWHI', 'BCUT2D_MWLOW', 'BCUT2D_CHGHI', 'BCUT2D_CHGLO', 'BCUT2D_LOGPHI', 'BCUT2D_LOGPLOW', 'BCUT2D_MRHI', 'BCUT2D_MRLOW', 'BalabanJ', 'PEOE_VSA1', 'PEOE_VSA2', 'PEOE_VSA3', 'PEOE_VSA4', 'PEOE_VSA5', 'PEOE_VSA6', 'PEOE_VSA7', 'PEOE_VSA8', 'PEOE_VSA9', 'PEOE_VSA10', 'PEOE_VSA11', 'PEOE_VSA12', 'PEOE_VSA13', 'PEOE_VSA14', 'MolMR', 'SMR_VSA1', 'SMR_VSA2', 'SMR_VSA3', 'SMR_VSA4', 'SMR_VSA5', 'SMR_VSA6', 'SMR_VSA7', 'SMR_VSA9', 'SMR_VSA10', 'VSA_EState1', 'VSA_EState2', 'VSA_EState3', 'VSA_EState4', 'VSA_EState5', 'VSA_EState6', 'VSA_EState7', 'VSA_EState8', 'VSA_EState9', 'VSA_EState10', 'NumAromaticRings', 'NumSaturatedRings', 'NumHeteroatoms'
